# Eculizumab in rapidly progressive glomerulonephritis: a case-series report

**DOI:** 10.3389/fimmu.2026.1919699

**Published:** 2026-09-28

**Authors:** Xueting Li, Yinghui Zhang, Ben Ke

**Affiliations:** Department of Nephrology, The Second Affiliated Hospital of Nanchang University, Nanchang, Jiangxi, China

**Keywords:** anti-glomerular basement membrane disease, anti-neutrophil cytoplasmic antibody-associated vasculitis, case series, eculizumab, rapidly progressive glomerulonephritis

## Abstract

**Objective:**

To describe the clinical outcomes and safety profile of eculizumab as adjunctive therapy in a case series of patients with rapidly progressive glomerulonephritis (RPGN) due to anti-glomerular basement membrane (GBM) disease and anti-neutrophil cytoplasmic antibody-associated vasculitis (AAV).

**Methods:**

We reported 7 patients with AAV and 5 patients with anti-GBM disease who presented to our center between March 2025 and January 2026. All patients received eculizumab (900 mg, 1–4 doses) in addition to standard immunosuppressive therapy. Serum creatinine (Scr), urinary red blood cell (uRBC) count, urine protein-to-creatinine ratio (UPCR), and dialysis dependence were assessed at baseline and at 3 months. Adverse events were monitored throughout follow-up.

**Results:**

In AAV patients, mean Scr decreased from 407.38 μmol/L to 231.27 μmol/L (*p* = 0.025), mean uRBC from 384.27/μL to 68.75/μL (*p* = 0.033), and mean UPCR from 2.20 g/g to 1.64 g/g (*p* = 0.179) at the 3rd month. In anti-GBM patients, mean Scr declined from 575.07 μmol/L to 263.80 μmol/L (*p* = 0.024), mean uRBC from 1648.60/μL to 461.92/μL (*p* = 0.132), and mean UPCR from 10.88 g/g to 3.75 g/g (*p* = 0.126). At baseline, 6 patients required renal replacement therapy, and after treatment, 4 became dialysis-independent. One AAV patient progressed to hemodialysis, and one anti-GBM patient remained dialysis-dependent. All anti-GBM antibodies and MPO-ANCA (except one patient) seroconverted to negative at month 3. During follow-up, only one case of COVID-19 infection and one oral ulcer were recorded; no severe infections occurred.

**Conclusion:**

In this descriptive case series, we observed trends toward improved renal function and hematuria at 3 months in patients with severe RPGN who received eculizumab in addition to standard immunosuppression, with earlier initiation and milder baseline renal impairment appearing to correlate with more favorable outcomes. Nevertheless, the lack of a control group and the marked interpatient variability in baseline characteristics and concomitant therapies preclude any conclusion regarding a causal effect of eculizumab. Instead, these data serve as real-world observations that can generate hypotheses for future studies. Definitive evidence on the efficacy and safety of eculizumab in RPGN must await larger, prospective, controlled investigations.

## Introduction

Rapidly progressive glomerulonephritis (RPGN) is a rare clinical syndrome characterized by acute loss of renal function, with renal pathology typically showing crescents in more than 50% of glomeruli. Based on the distribution of immune deposits as visualized by immunofluorescence and electron microscopy, RPGN can be classified into three types: anti-glomerular basement membrane (GBM) disease, immune complex glomerulonephritis, and pauci-immune glomerulonephritis, the last of which is typically associated with antineutrophil cytoplasmic antibodies (ANCAs) (1). Without timely and effective treatment, RPGN often progresses to end-stage renal disease within weeks to months. Current standard treatment options for RPGN include glucocorticoids, cyclophosphamide (CTX), rituximab (RTX), and plasma exchange (PE) (2, 3). Nevertheless, a considerable proportion of patients fail to achieve remission and eventually progress to end-stage renal disease (ESRD) (4), indicating that existing therapies do not meet clinical needs and that novel treatment strategies are urgently required.

Abnormal complement activation plays a critical role in the pathogenesis of both anti-GBM disease and anti-neutrophil cytoplasmic antibody-associated vasculitis (AAV) (5). In anti-GBM disease, binding of anti-GBM antibodies to the glomerular basement membrane potently activates complement via the classical pathway, leading to the formation of membrane attack complexes that directly damage the glomerular capillary wall and promote crescent formation. In AAV, ANCA antibodies bind to antigens on the surface of activated neutrophils, activating complement via the alternative pathway and generating large amounts of C5a, which in turn chemoattracts and activates neutrophils, establishing a positive feedback loop that accelerates glomerular necrosis and crescent formation (6). Given the central role of complement in both diseases, the use of complement inhibitors in RPGN has garnered considerable attention. The ADVOCATE trial provided promising evidence for the oral C5a receptor antagonist avacopan in AAV, leading to its regulatory approval in some regions (7). However, avacopan is not approved for anti-GBM disease, and its use in patients with severe renal impairment remains inadequately characterized. In contrast, eculizumab is a humanized monoclonal antibody targeting C5, which can block the cleavage of C5 and inhibit the formation of C5a and C5b-9, and has only been suggested in case reports or small case series to have potential therapeutic benefit in anti-GBM disease or AAV, and current clinical evidence remains very limited (8, 9).

In this case series, we aimed to report our real-world experience with the addition of eculizumab to conventional therapy in patients with severe RPGN due to anti-GBM disease and AAV.

## Patients and methods

This single-center retrospective case series included 12 patients diagnosed with rapidly progressive glomerulonephritis (RPGN) between March 2025 and January 2026, comprising 7 patients with anti-neutrophil cytoplasmic antibody-associated vasculitis (AAV) and 5 patients with anti-glomerular basement membrane (GBM) disease. All patients were diagnosed and treated at the Department of Nephrology, The Second Affiliated Hospital of Nanchang University.

Inclusion criteria were: (i) a confirmed diagnosis of AAV or anti-GBM disease based on serology or renal biopsy pathology; (ii) receipt of eculizumab as adjunctive therapy in addition to standard immunosuppressive treatment; and (iii) availability of baseline and 3-month follow-up data.

Exclusion criteria were: (i) active infection; (ii) malignancy of any type; (iii) known hypersensitivity to eculizumab; (iv) pregnancy or lactation.

### Statistical analysis

Continuous variables are presented as mean (range) due to the small sample size. Given the paired nature of the data, comparisons of serum creatinine, urinary red blood cell count, and urine protein-to-creatinine ratio before and after treatment were performed using paired t-tests or Wilcoxon signed-rank tests, depending on the results of normality testing. Normality was assessed using the Shapiro–Wilk test; if the assumption of normality was met and variances were homogeneous, paired t-tests were applied; otherwise, Wilcoxon signed-rank tests were used. Statistical analyses were conducted using SPSS software (version 26.0; IBM SPSS Company, Chicago, Illinois). A two-sided p < 0.05 was considered statistically significant.

## Results

A total of 12 patients were included in this case series, comprising 7 patients with AAV and 5 patients with anti-GBM disease. The mean age of patients with AAV was 68 years, with 4 males and 3 females. Among the patients with AAV, P1 had comorbid alcoholic liver cirrhosis, P5 had hypothyroidism, and P6 had hepatitis B and type 2 diabetes mellitus; the remaining patients had no documented comorbidities. The median time from first symptom onset to initiation of eculizumab therapy in AAV patients was 2.0 months. One patient (P2) had an interval of up to 8 months, during receiving methylprednisolone (MP) pulse therapy, CTX, RTX, PE, and required renal replacement therapy.

The mean age of anti-GBM patients was 30.8 years, with 2 males and 3 females. None of the anti-GBM patients had any comorbidities. The median time from first symptom onset to eculizumab initiation in anti-GBM patients was 1.9 months. One patient (P12) had an interval of up to 6 months, during receiving MP pulse therapy and PE, but the disease did not achieve remission.

All patients with RPGN received eculizumab in addition to immunosuppressive therapy (Corticosteroids, CTX, RTX). The single dose of eculizumab was 900 mg, administered 1 to 4 times. All patients received prophylactic cefdinir to prevent infections, particularly Neisseria meningitidis. The clinical profiles and treatment courses of the 12 patients are detailed in **Table 1** and **Figure 1A**.

**Table 1:** Clinical features of the patient presentation across cases.

Characteristic	P1	P2	P3	P4	P5	P6	P7	P8	P9	P10	P11	P12
Features on admission												
Age, years	59	71	73	65	88	61	59	23	33	30	37	31
Gender	M	M	F	M	F	F	M	F	M	F	M	F
Context	AAV	AAV	AAV	AAV	AAV	AAV	AAV	anti-GBM nephritis	Atypical anti-GBM nephritis	anti-GBM nephritis	anti-GBM nephritis	anti-GBM nephritis
Comorbidities	Alcoholic liver cirrhosis	N/A	N/A	N/A	Hypothyroidism	hepatitis B diabetes	N/A	N/A	N/A	N/A	N/A	N/A
Interval between 1st symptoms to the initiation of eculizumab treatment, months	2	8	0.33	0.5	4	2	1	0.5	0.5	1.5	1	6
Prior treatment	MP40mg*6d	MP40mg/qd CTX0.2g/qod PE*5 RTX600mg*2	No	MP20mg/qd	MP40mg/qd	No	No	No	No	No	No	PE*5 MP40mg/qd
RRT	No	HD	No	No	No	No	HD	HD	HD	HD	No	HD
Scr, μmol/l	243	221	375	634	356	412.28	610.41	529	595	736	247	768.36
eGFR (mL/min/1.73 m^2^)	24	17.9	10.3	4.6	9.4	10.14	10.1	9.2	9.8	5.8	26.6	5.71
uPCR(g/g)	3.61	0.28	1.12	6.3	2.7	0.99	0.37	6.64	8.31	8.58	5.34	25.54
Albumin (g/L)	25.9	35.6	29.1	27.3	29.8	30.47	28	34.6	22.4	24.6	34	45.09
Hb(g/L)	79	98	86	71	97	76	50	109	108	83	144	78
uRBC,/μl	530	6.5	26.5	568	810.6	395.7	352.6	1491.8	386.7	4166.4	1668.1	530
anti-GBM, RU/ml	1.27	13.62	1.84	0.85	0.95	212.8	52.40	185.99	9.13	55.85	56.6	>400
anti-MPO, RU/ml	94.26	7.31	>300	100.58	14.11	986.4	247.1	1.2	2.46	2.04	N/A	1.8
IgA, mg/dl	265.2	112	269.2	169.8	202.2	411	165	140.5	208.9	250.8	126.6	132
IgM, mg/dl	157.1	73.5	46.3	66.7	120.3	95.7	157	112	52.6	148.4	76.8	66.3
IgG, mg/dl	1119	1260	2006	1197	1013	1700	1795	859	887	778	599	430
C3, mg/dl	142.7	107	161.3	101.1	101.8	N/A	157	109.3	160.2	110.3	137.2	75.2
C4, mg/dl	42.8	44.7	36.8	27	27.4	N/A	16.5	31.9	46.6	25	36.1	20.2
Treatment after admission (d0)												
Other treatment	RTX600mg*2 CTX0.4g pred30mg/qd	MP40mg/qd	MP250mg*3d RTX400mg*3	MP500mg*3d RTX300mg*2	MP20mg/qd RTX100mg	MP500mg*3d PE*5 CTX0.4g RTX300mg	MP300mg*3d PE*5 RTX300mg CTX0.4g	MP500mg*3d CTX0.2g/qod(1.8g) RTX600mg	MP500mg*3d MP80mg*8d CTX0.2g/qod(1.0g) Obinutuzumab1000mg Zebutumab200mg	MP500mg*3d MP80mg*10d CTX0.2g/qod(1.4g) Zebutumab800mg	MP500mg*3d RTX300mg MP40mg/qd	MP40mg/qd CTX0.6*6 RTX1000mg*2 PE*5
Eculizumab	900mg*2 (d0,d20)	900mg (d0)	900mg*4 (d0,d7,d30,d60)	900mg*2 (d0,d7)	900mg*2 (d0,d6)	900mg (d0)	900mg (d0)	900mg*4 (d0,d7,d14,d21)	900mg*2 (d0,d8)	900mg*3 (d0,d7,d14)	900mg*4 (d0,d14,d27,d43)	900mg*3 (d0,d7,d14)
Outcome in the first months												
RRT dependent	No	No	No	HD	No	No	No	No	No	PD	No	HD
Scr, μmol/l	162	208	313	920	297	185.38	250.95	218	332	725	181	966.25
uRBC,/μl	477.5	2.6	1.7	278.9	199.4	157.8	225.2	1251.2	217	1660.9	300.5	477.5
anti-GBM, RU/ml	N/A	1.33	N/A	N/A	N/A	N/A	N/A	N/A	15.1	20.69	3.96	24.1
anti-MPO, RU/ml	58.94	2.72	38.58	54.45	N/A	45.8	88.1	73.98	N/A	N/A	N/A	N/A
IgA, mg/dl	133.9	94.9	N/A	N/A	N/A	N/A	226	75.1	132.8	131.8	N/A	134
IgM, mg/dl	148.1	55.8	N/A	N/A	N/A	N/A	722	35.2	49.8	102.3	N/A	71.1
IgG, mg/dl	571	457	N/A	N/A	N/A	N/A	760	74	444	187	N/A	680
C3, mg/dl	116.4	109.6	N/A	N/A	N/A	N/A	72.2	65	137.9	56	N/A	N/A
C4, mg/dl	33.2	32.4	N/A	N/A	N/A	N/A	22.6	18	51.1	5.4	N/A	N/A
Outcome in the 3rd month												
RRT	No	No	No	HD	No	No	No	No	No	PD	No	HD
Scr, μmol/l	138	191	285	409	276	88.66	N/A	169	310	127	177	535.99
uRBC,/μl	60.8	2.4	2.6	169.8	107.3	69.6	N/A	1169.8	109.1	664.2	199.4	167.1
anti-GBM, RU/ml	N/A	1.74	N/A	N/A	N/A	N/A	N/A	N/A	1.32	3.68	4.44	6.6
anti-MPO, RU/ml	N/A	1.82	9.45	32.02	N/A	5.9	N/A	N/A	N/A	N/A	N/A	N/A
IgA, mg/dl	N/A	105	112.9	90.4	N/A	N/A	N/A	N/A	N/A	94.8	N/A	104
IgM, mg/dl	N/A	62.6	24.6	37.4	N/A	N/A	N/A	N/A	N/A	71.4	N/A	62.2
IgG, mg/dl	N/A	535	623	854	N/A	N/A	N/A	N/A	N/A	179	N/A	490
C3, mg/dl	N/A	119.4	100.4	82.9	N/A	149	N/A	N/A	N/A	106.2	N/A	N/A
C4, mg/dl	N/A	37.4	30.8	26.9	N/A	45.4	N/A	N/A	N/A	24.1	N/A	N/A
Long-term outcome												
Follow-up, months	3	5	4	10	3	5	3	7	3	3	9	5
Scr at the last follow-up, μmol/l	162	189	218	794	297	106.6	212.49	137	310	127	181	302.6
RRT dependent	No	No	No	HD	No	No	No	No	No	PD	No	HD
Maintenance treatment	pred	Pred/RTX	pred	pred	pred	CTX/Triamcinolone	Triamcinolone	pred	MP	MP	pred	Pred/RTX
Complication							Oral ulcer					

RRT, renal replacement therapy; N/A, not applicable; pred, prednisone; CTX, cyclophosphamide; RTX, rituximab; PE, plasma exchange; HD, Hemodialysis;PD, Peritoneal dialysis.

**Figure 1:**
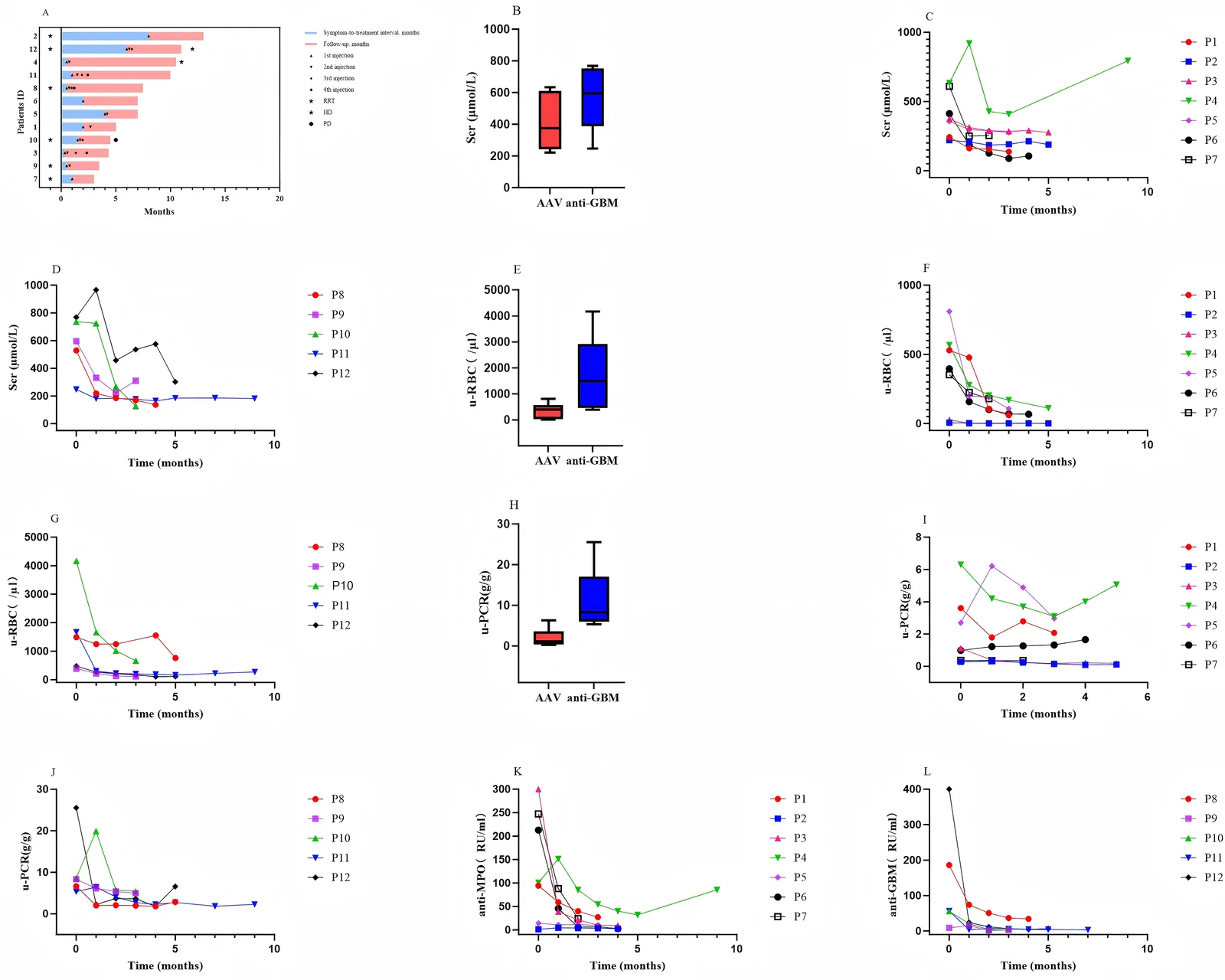
Changes in clinical indicators during follow-up. **(A)** Flowchart of the diagnosis and treatment process for 12 patients with RPGN. **(B, E, H)** Box plots illustrating the overall distributions and general profiles of serum creatinine (Scr), urine red blood cell (uRBC) counts, and urine protein-to-creatinine ratio (uPCR) in the AAV and anti-GBM groups, respectively, providing a direct visual overview of the distinct laboratory characteristics between the two diseases. **(C, D)** Trends in Scr during follow-up. **(F, G)** Trends in uRBC during follow-up. **(I, J)** Trends in uPCR during follow-up. **(K)** Trends in anti-MPO antibody levels in Cases 1–7. **(L)** Trends in anti-GBM antibody levels in Cases 8–12.

At the first dose of eculizumab, the mean serum creatinine (Scr) level in AAV patients was 407.38 μmol/L (range, 221–634 μmol/L), the mean urinary red blood cell (uRBC) count was 384.27/μL (range, 6.5–810.60/μL), and the mean urine protein-to-creatinine ratio (UPCR) was 2.20 g/g (range, 0.28–6.30 g/g). Among the AAV patients, two required renal replacement therapy upon admission to our center; both became dialysis-independent after treatment. However, one patient (P4) experienced worsening renal function despite high-dose MP pulse therapy, RTX, and eculizumab, eventually progressing to hemodialysis. At 3 months, their mean Scr decreased from 407.38 μmol/L to 231.27 μmol/L (*p* = 0.025); their mean uRBC decreased from 384.27/μL to 68.75/μL (*p* = 0.033); and their mean UPCR decreased from 2.20 g/g to 1.64 g/g (*p* = 0.179) (**Figure 1**). Except for P4, all AAV patients tested negative for anti-myeloperoxidase (MPO) antineutrophil cytoplasmic antibodies at month 3.

At the first dose of eculizumab, the mean Scr level in anti-GBM patients was 575.07 μmol/L (range, 247–768.36 μmol/L), the mean uRBC count was 1648.60/μL (range, 386.70–4166.40/μL), and the mean UPCR was 10.88 g/g (range, 5.34–25.54 g/g). Among the anti-GBM patients, four required renal replacement therapy upon admission to our center; after treatment, two became dialysis-independent, one was switched from hemodialysis to peritoneal dialysis, and one remained dialysis-dependent. At 3 months, their mean Scr decreased from 575.07 μmol/L to 263.80 μmol/L (*p* = 0.024); their mean uRBC decreased from 1648.60/μL to 461.92/μL (*p* = 0.132); and their mean UPCR decreased from 10.88 g/g to 3.75 g/g (*p* = 0.126) (**Figure 1**). Furthermore, all anti-GBM antibodies tested negative at month 3. During follow-up, no severe infections occurred in the remaining patients except for one case of COVID-19 infection (P4) and one case of oral ulcer (P7).

## Discussion

In our case series, six patients required renal replacement therapy upon admission. After receiving immunotherapy combined with eculizumab, four of them successfully discontinued hemodialysis, while the remaining two remained dependent on renal replacement therapy. Notably, among the two patients who did not achieve dialysis independence, P10 had a high pre-treatment Scr level (736 μmol/L), whereas P12 had a delay of up to six months from the onset of initial symptoms to the initiation of eculizumab treatment, and a high level of anti-GBM antibodies (>400 RU/mL) at diagnosis. These findings are consistent with existing studies, indicating that severe impairment of baseline kidney function, high antibody levels, and delayed treatment initiation affect renal outcomes (10). Furthermore, the efficacy of eculizumab may also be closely related to the timing of treatment and baseline renal function. Therefore, early initiation of therapy for RPGN is crucial to prevent disease progression to kidney failure.

In the treatment of RPGN, CTX, RTX, and glucocorticoids primarily act by inhibiting B-cell and T-cell function and reducing autoantibody production, thereby blocking the source of the pathogenic immune response. However, even when antibody production is effectively suppressed, the already activated complement cascade can continue to drive downstream tissue injury. In this setting, the addition of eculizumab for the treatment of RPGN may be considered. Current evidence indicates that eculizumab directly targets complement C5 and, within a short time after infusion, completely blocks the terminal complement pathway, rapidly inhibiting C5a-mediated inflammatory chemotaxis and tissue damage caused by the C5b-9 membrane attack complex (11). This quickly curtails necrotizing inflammation in the glomeruli. Therefore, combining eculizumab with standard upstream immunosuppressive therapy achieves a synergistic effect: upstream inhibition of autoantibody production and downstream rapid blockade of complement-mediated injury. This approach is particularly suitable for patients with rapidly progressive glomerulonephritis who require prompt control of active inflammation.

In summary, this descriptive case series provides real-world observations that may inform future research. The addition of eculizumab to standard immunosuppression and plasma exchange was associated with encouraging trends in renal function and urinary parameters in some patients with severe RPGN, particularly those with earlier treatment initiation and better baseline renal function. However, given the uncontrolled design, small sample size, and substantial interpatient heterogeneity, these observed changes cannot be attributed specifically to eculizumab. The role of eculizumab in RPGN warrants rigorous evaluation in adequately powered, prospective, controlled trials, and its clinical use should be individualized based on renal function, antibody titers, renal biopsy findings, and timing of treatment initiation.

## Data Availability

The raw data supporting the conclusions of this article will be made available by the authors, without undue reservation.
